# Rhabdomyosarcomas in Aging A/J Mice

**DOI:** 10.1371/journal.pone.0023498

**Published:** 2011-08-10

**Authors:** Roger B. Sher, Gregory A. Cox, Kevin D. Mills, John P. Sundberg

**Affiliations:** The Jackson Laboratory, Bar Harbor, Maine, United States of America; Medical College of Georgia, United States of America

## Abstract

Rhabdomyosarcomas (RSCs) are skeletal muscle neoplasms found in humans and domestic mammals. The A/J inbred strain developed a high frequency (between 70–80%) of adult pleomorphic type (APT) RSC at >20 months of age while BALB/cByJ also develop RSC but less frequently. These neoplasms invaded skeletal muscle surrounding either the axial or proximal appendicular skeleton and were characterized by pleomorphic cells with abundant eosinophilic cytoplasm, multiple nuclei, and cross striations. The diagnosis was confirmed by detection of alpha-sarcomeric actin and myogenin in the neoplastic cells using immunocytochemistry. The A/J strain, but not the related BALB/c substrains, is also characterised by a progressive muscular dystrophy homologous to limb-girdle muscular dystrophy type 2B. The association between the development of RSC in similar muscle groups to those most severely affected by the progressive muscular dystrophy suggested that these neoplasms developed from abnormal regeneration of the skeletal muscle exacerbated by the dysferlin mutation. Transcriptome analyses of RSCs revealed marked downregulation of genes in muscular development and function signaling networks. Non-synonymous coding SNPs were found in *Myl1, Abra, Sgca, Ttn,* and *Kcnj12* suggesting these may be important in the pathogenesis of RSC. These studies suggest that A strains of mice can be useful models for dissecting the molecular genetic basis for development, progression, and ultimately for testing novel anticancer therapeutic agents dealing with rhabdomyosarcoma.

## Introduction

While rhabdomyosarcoma (RSC) represent 50% of all soft tissue sarcomas and ∼10% of all malignant solid tumors in children [1], adult pleomorphic RSCs are relatively rare. The average annual incidence of RSC is ∼4 cases per million in the US in children aged 0–14 [2], with >50% of all RSCs diagnosed in children <10 years of age [3]. RSCs are classified into three distinct histopathologic subtypes: embryonal (ERSC) constituting ∼80% of cases, alveolar (ARSC) constituting ∼20% of cases, and the adult pleomorphic type (APT) occurring in ∼3% of cases [4], [5]. APT predominantly affects the extremities, is usually chemoresistant and is associated with poor prognosis [5]. Several animal models for ERSC and ARSC have been developed through genetic manipulations, but spontaneous models similar to the adult onset human APT are lacking.

Studies using animal models to establish the processes involved in skeletal muscle development have been crucial to the investigation of abnormalities in skeletal muscle development such as RSC, congenital muscular dystrophy, and congenital myopathy [6]. RSCs rarely occur in laboratory mice in large production colonies maintained up to an average of 8 months of age, but a susceptibility to the development of spontaneous RSCs was identified in BALB/cJ and BALB/cByJ strains and in other closely related strains [7], [8]. In confirmation of this strain effect, development of RSC in the patched homolog 1 (*Ptch1^tm1Zim^*) mouse model for Gorlin syndrome, which results in RSC and other tumors in humans, strongly depends on the genetic background with BALB/c being a susceptible strain for RSC development and C57BL/6 showing resistance [9], [10]. In the course of an ongoing mouse aging study at The Jackson Laboratory [11], [12] we found that the A/J mouse strain develops APT RSC at a high frequency over 20 months of age, which confirmed our earlier strain disease surveillance studies. This strain is also characterized by a progressive muscular dystrophy homologous to limb-girdle muscular dystrophy type 2B, due to a retrotransposon insertion in the dysferlin gene [13]. Two recent reports found that mice with mutations in the dystrophin-associated glycoprotein (DAG) complex (dystrophin, muscular dystrophy; X linked muscular dystrophy null (*Dmd^mdx^*) and alpha-sarcoglycan-null, B6.129S6-*Sgca^tm2Kcam^/*J also develop spontaneous rhabdomyosarcomas, despite being on generally non-susceptible backgrounds (C57BL/10J and C57BL/6J, respectively) [14], [15]. This may indicate that underlying skeletal muscle disease could predispose muscles to development of RSC.

The importance of developing new animal models for therapeutic testing is illustrated by the recent report of inhibition of RSC development in Patched (*Ptch^tm1Zim^/+*) heterozygous mice (which develop medulloblastoma and RSC) with DNA methyltransferase1 and histone deacetlyase inhibitors [16]. Additional animal models provide new insight into the biochemical pathways responsible for the development of RSC. Here, we show that A strains of mice are susceptible to APT RSC development and we describe a new model for spontaneous rhabdomyosarcoma.

## Results

### Mice

Retrospective examination of medical records of all A/J mice submitted for either necropsy or research post mortem examination over a 15 year period [8], [17], [18] identified the frequency of APT RSC diagnosis was 17/1017 (1.7%). In the closely related BALB/cJ and BALB/cByJ mice, RSC was diagnosed in 22/2089 BALB/cJ (∼1%) & 11/1321 BALB/cByJ (∼0.8%) mice that were submitted for either diagnostic or research post mortem examination. These numbers are deceptive in that they represent data from disease surveillance of production colonies that were rarely maintained past 8–12 months of age. These results confirm that BALB/c substrains and A/J are susceptible strains and that RSC naturally occur late in life. No cases of rhabdomyosarcoma were diagnosed in 129S1/SvlmJ, AKR/J, BTBR-T+tf/J, BUB/BnJ, C3H/HeJ, C57BL/6J, C57BL/10J, C57BLKS/J, C57BR/cdJ, C57L/J, CBA/J, DBA/2J, FVB/NJ, KK/H1J, LP/J, MRL/J, NON/LtJ, NZO/H1LtJ, NZW/LacJ, P/J, PL/J, PWD/PhJ, RIIIS/J, SJL/J, SM/J, SWR/J, or WSB/EiJ mice in the aging study [11], [12].

We found that in the aging study population under detailed investigation the A/J mice developed APT RSC at a relatively high frequency in mice over 20 months of age (Table 1). By contrast, only one case was diagnosed in a 20 month old male BALB/cByJ mouse. None of the SJL/J mice, which also carry a mutant allele of dysferlin, developed RSC; however, many fewer old mice of this strain were obtained primarily due to fighting and their propensity for other types of cancer, including spontaneous reticulum cell sarcomas [19].

**Table 1:** Frequency of APT rhabdomyosarcomas by strain and gender.

Age	A/J						BALB/cByJ						SJL/J					
	Females			Males			Females			Males			Females			Males		
	Rhab	Total	%	Rhab	Total	%	Rhab	Total	%	Rhab	Total	%	Rhab	Total	%	Rhab	Total	%
6 mo.	0	10	0	0	9	0	0	10	0	0	10	0	0	10	0	0	8	0
12 mo.	1	14	7	0	13	0	0	12	0	0	14	0	0	23	0	0	2	0
20 mo.	1	7	14	1	9	11	0	15	0	1	9	11	0	0	0	0	0	0
>20 mo.	15	19	79	7	10	70	0	12	0	0	4	0	0	13	0	0	8	0
Total	17	50	34	8	41	20	0	51	0	1	37	3	0	46	0	0	18	0

### Histopathology and Immunocytochemistry

**Figure 1:**
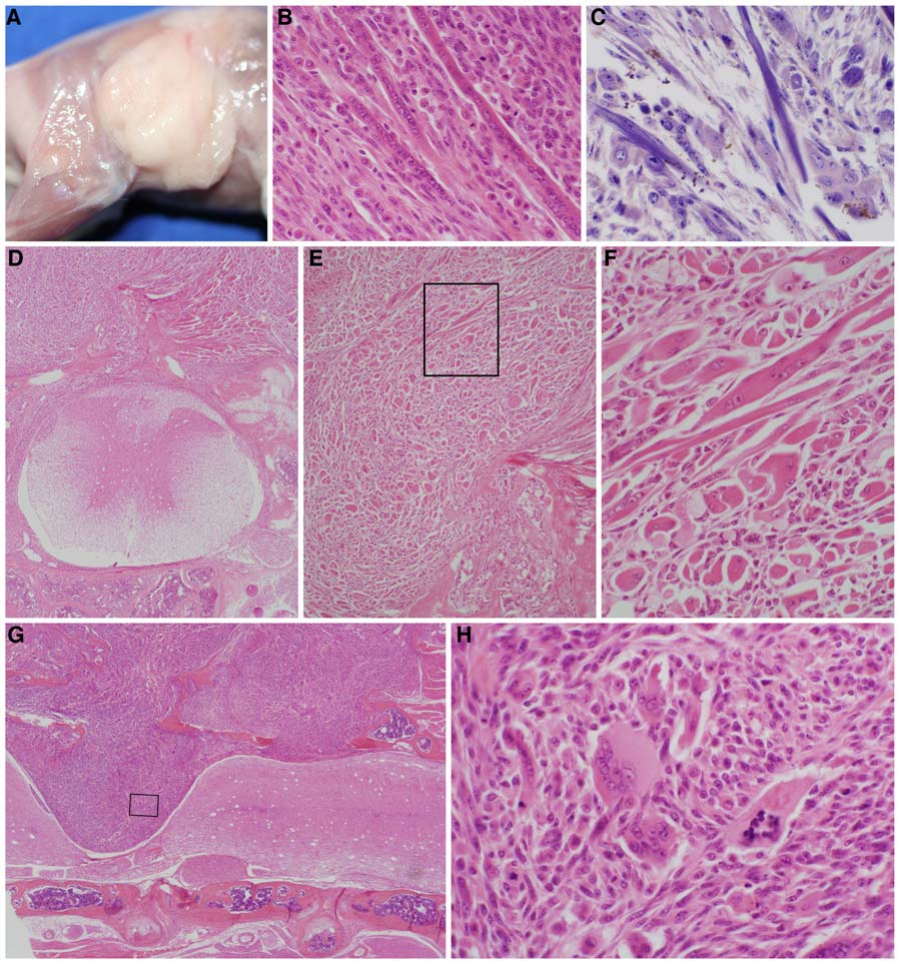
Rhabdomyosarcoma in A/J mice >500d. (A) A large, pale, irregular mass in the lumbar region was firmly attached to the underlying muscle in this moribund A/J mouse. (B) Neoplastic cells were small, round cells with a single nucleus or elongated strap-like cells with regimented clusters of nuclei (H&E). (C) Cross striations were evident in the strap-like cells when sections were stained with phosphotungstic acid hematoyxlin. (D) Bones and normal skeletal muscle were infiltrated by the neoplastic cells above the vertebrae and spinal cord, destroying the normal architecture. (E) Higher magnification of A demonstrated the pleomorphic phenotype of the APT RSC. (F) Higher magnification of the boxed area in E illustrated strap-like, spindle-shaped, and single round cells with abundant brightly eosinophilic cytoplasm (H&E). (G) APT RSC invaded locally through the bone and compressed the spinal cord. (H) Higher magnification of boxed area illustrated the poorly differentiated multinucleated giant cells (arrows) and a bizarre mitotic figure (boxed area; H&E).

Aging A/J mice developed APT RSC. These appeared as firm, irregular masses on the dorsal or lateral surfaces that were attached to the underlying musculature. These neoplasms were pale tan when the skin was removed (Fig. 1a). The tumors were often multifocal and locally invasive separating adjacent muscle bundles. Microscopically, the tumor consisted of pleomorphic round to spindle shaped cells, depending on orientation, with single or multiple vesicular nuclei and one or more prominent nucleoli. The morphology of these neoplastic cells did not resemble either ARSC or ERSC. Cytoplasm was brightly eosinophilic (Fig. 1b). Cross striations were evident with special stains (Fig. 1c). Rhabdomyosarcomas often arose from the epaxial muscles over the lumbar vertebrae (Fig. 1d–f). Aggressive lesions would extend ventrally to completely efface the locally affected vertebrae sometimes compressing the spinal cord (Fig. 1g). These aggressive cancers were very pleomorphic with abundant and often bizarre mitotic figures (Fig. 1 h). The solitary RSC in the BALB/cByJ mouse was well differentiated but locally aggressive as previously reported for this strain [8].

**Figure 2:**
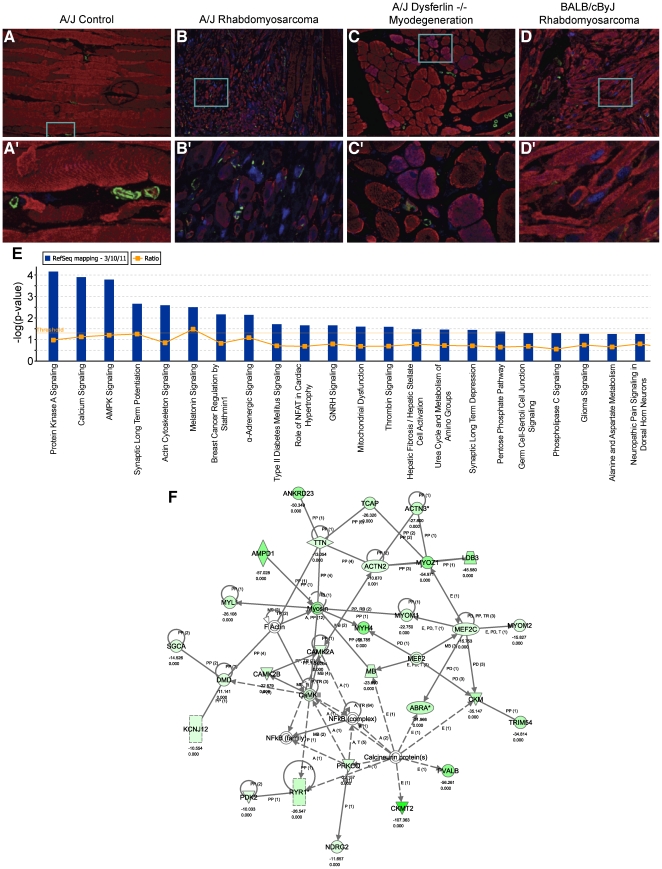
Protein expression in mouse rhabdomyosarcomas and Ingenuity Pathway Analysis of Illumina microarray data. (A–D) Immunohistochemistry confirmed the tumor cells contained sarcomeric actin and myogenin but not smooth muscle actin, thereby confirming the diagnosis of rhabdomyosarcoma. A–D, 20x; A’–D’ boxed areas from A–D at 63x. Red = anti-sarcomeric actin, Green = anti-smooth muscle actin, Blue = anti-myogenin. (E) Signalling pathways of the most significantly dysregulated networks when a 10 fold or greater change was used as a cutoff. (F) Most of the genes in this network were significantly downregulated (green).

The APT RSCs were consistently positive for striated muscle actin isoform but expression by immunofluorescence was reduced compared to that in normal skeletal muscle samples used as controls (Fig. 2a–d). In addition, APT RSC, but not normal skeletal muscle, was positive for myogenin (Fig 2a–d). Smooth muscle actin isoform expression was limited to vascular smooth muscle walls (Fig. 2a–d).

### Gene Expression Studies

To identify the underlying molecular pathways and potential candidate genes for rhabdomyosarcoma, we used Illumina Mouse BeadChip gene arrays to compare transcript levels (Geo_Accession: GSE29775) between 4 rhabdomyosarcomas (∼450 day old male A/J mice) and 8 normal skeletal muscle samples (425–550 day old male A/J mice). Relative fold change for each gene was determined in APT RSC samples relative to normal skeletal muscle samples. Using Ingenuity Pathway Analysis® software, with a cut off of 10 fold or greater change, we identified the major dysregulated molecular pathways (Fig. 2e). Of particular note are the calcium, AMPK, and actin cytoskeleton-signalling pathways because they predicted significant down regulation of many terminal differentiation proteins (Fig. 2f), which was evident in the immunocytochemistry (Fig. 2a–d).

Scanning the most significantly downregulated genes (green and dark green in Fig. 2f) using the Sanger SNPs database (http://www.sanger.ac.uk/cgi-bin/modelorgs/mousegenomes/snps.pl) revealed that *Actn2*, *Dmd*, *Actn3*, *Tcap*, *Myoz1*, *Ampd1*, *Myh4*, *Ldb3*, *Camk2b*, *Ankrd23, Mef2c, Myom2, Mef2, Mb, CamkII, Ckm, Trim54, Pvalb, Ckmt2, Prkcq, Pdk2*, and *Camk2a* had no non-synonymous coding SNPs (nsSNP). *Myom1* had 5 nsSNPs that it shared with BALB/cJ and several other strains and *Ryr1* shared 1 nsSNP exclusively with BALB/cJ and 1 nsSNP it shared with BALB/cJ and other strains. Alpha sarcoglycan (dystrophin-associated glycoprotein) (*Sgca*) had one nsSNP it shared with several other strains (not BALB/cJ) (7 targeted mutants, all with muscular dystrophy, listed in Mouse Genome Informatics (http://www.informatics.jax.org/)), *Myl1* had a lost stop codon it shared with several other strains (not BALB/cJ) (3 targeted mutants, one with muscle defects, one with growth defects; MGI), *Abra* had 4 nsSNPs it shared with several other strains (not BALB/cJ) (1 targeted mutant with cardiovascular defects; MGI), Titin (*Ttn*) had one nsSNP in A/J that it did not share with any other strain (6 spontaneous, chemically-induced, and targeted mutants, all with muscular dystrophy/defects; MGI), and the potassium inwardly rectifying channel, subfamily J, member 12 (*Kcnj12*) had 3 nsSNPs all marked as having multiple consequences that it shared with several other strains (not BALB/cJ; no evidence of skeletal muscle problems with 2 targeted mutants; MGI). As mentioned previously, B6.129S6-*Sgca^tm2Kcam^/*J null mice also develop random RSCs, despite being on a non-permissible background (C57BL/6J) [14]. Therefore, these genes (*Myl1, Abra, Sgca, Ttn, Kcnj12*) and proteins will be the focus of future investigations.

## Discussion

A number of clinically relevant cancers regularly arise in some inbred mouse strains but not in others. Two strains, A/J and SJL/J, are known to develop muscular dystrophy with age due to null mutations in the dysferlin gene (*Dysf*) [13], [20]. We analyzed areas of abortive skeletal muscle regeneration in these two strains and, surprisingly, observed that rhabdomyosarcomas (aggressive malignancies of skeletal muscle) develop in A/J but not SJL/J mice (for additional images go to http://www.pathbase.net/and http://tumor.informatics.jax.org/). BALB/cByJ mice, (wildtype for *Dysf* and related by decent to A/J mice [21]), develop rhabdomyosarcomas [8], but as shown here, less frequently than A/J in older populations. Histochemical and immunocytochemical studies of lesions from A/J and BALB/cByJ mice confirmed that these were skeletal muscle neoplasms. Chamberlain *et al.* [15] in discussing the development of RSCs in the dystrophin-null *Dmd^mdx^* mouse strain, speculated that “the lifelong continuous myofiber degeneration and regeneration that characterize this animal model are associated with continuous and massive activation and proliferation of satellite cells, which greatly increases the chance of developing random and spontaneous mutations.” Although mutations in muscle-related genes may be associated with RSC development, as stated, genetic background can have a strong impact on whether these strains produce tumors. This knowledge will open up future genetic analysis of modifier genes for the development of RSCs.

Development of spontaneous RSCs in the A/J and less frequently in the related BALB/c substrains (Group 1 by descent from Bagg albino [21]) indicates that these strains are susceptible to RSCs. The additional burden of lifelong muscular dystrophy may increase frequency and accelerate oncogenesis, as speculated by Chamberlain *et al.* [15] but this is not supported by the failure to detect RSC development in the SJL/J strain, a non-related, Group 2 strain [21] with a dysferlin splice site mutation that removes part of the C2E domain [20]. This may require aging large numbers of SJL/J mice to validate this observation as many died before reaching 20 months of age due to unrelated diseases. A similar background effect was reported due to the activation of the *HER-2/neu* oncogene coupled with inactivation of *Trp53*, which resulted in RSC development in the BALB/cJ strain [22] but not the FVB/NHd strain (closely related to SJL/J), which only develop salivary tumors [23]. In addition, we have found that the A/J and related strains are extremely low in their apoptotic response (as tested in peripheral blood mononuclear cells, erythrocytes, and erythrocyte precursors) while strains more closely related to SJL/J rank among the highest in apoptosis rates (data not shown, http://phenome.jax.org/), indicating that strain differences in apoptosis may be relevant to the development and/or progression of RSCs. Indeed, it has been shown that down-regulation of apoptosis through PAX-FKHR fusion [24] and through *Trp53/Fos* double mutants enhances RSC tumor cell proliferation and invasion and contributes to the formation of RSCs.[25]

Molecular studies of human RSC identified both common and distinct molecular alterations occurring in the two main subtypes of RSC. Chromosomal translocations between forkhead transcription factor (*FKHR*) and either the *PAX3* (t2;13)(q35;q14) and *PAX7* (t1;13)(p36;q14) genes are found in the majority of ARSC [26] resulting in chimeric PAX3-FKHR and PAX7-FKHR transcription factors. A mouse model for ARSC was developed through a conditional knock-in allele of *Pax3:Fkhr* in the *Pax3* locus and this model gives a good reproduction of human ARSC [27]. A mouse model of APT RMS was developed through conditional activation of *K-rasG12V* in *Trp53^tm1Sia^* mice [28]. In contrast, ERSCs generally have a loss of heterozygosity (LOH) at the 11p15 region, resulting in increased expression of several imprinted genes such as *H19*, *IGF2*, and *p57^KIP2^* [29], [30], loss of skeletal muscle expression of the normally highly expressed gene *GOK* [31], and potentially the loss of a tumor suppression gene located in the region [32].

Early animal models for RSC go back to the 1950's, with viral and chemical agents used for tumor induction [33]. These RSC models are important for understanding the underlying genetics resulting in RSC initiation, for the identification of development of tumor growth, and for evaluation of therapeutic interventions [33]. RSCs are thought to arise from imbalances in cell proliferation (failure to exit the cell cycle) and differentiation (failure to differentiate into skeletal muscle) [34], [35]. Evidence for this includes overexpression of the muscle-specific microRNA miR-206 blocking RSC tumor growth by promoting the global gene profile toward mature muscle differentiation [36]. In addition, overexpression of myogenin in the RD/12 human RSC cell line reduces cell migration and tumor growth in mice [37]. Recently, it was also reported that a deficit in mitochondrial biogenesis and/or metabolism may contribute to the failure of RSCs to exit the cell cycle and differentiate [38].

The two main types of RSCs are thought to develop from distinct cell populations, with ERSCs developing from muscle progenitors and ARSCs from mesenchymal progenitor cells [39]. Even though the exact cell type(s) of origin are currently under debate, all RSCs exhibit some markers of embryonic or adult myogenic origin [33] and evidence points to the muscle stem cell known as the satellite cell [40], especially for ERSC. Satellite cells reside in a quiescent state under the basil lamina, and are rapidly activated and progress through an asymmetrical cell division upon injury to muscle tissue [41]. Some subpopulation of the activated satellite cells undergo differentiation into fused myotubes while others return to the quiescent state, replenishing the pool of satellite cells [42]. The role of PAX3 and PAX7 in the development of RSCs may reflect an exaggeration of the normal function of these genes to keep satellite cells in a proliferative-ready state, thus enabling rapid muscle repair [43]. In this context, it was recently reported that aberrantly regulated proliferative responses in mesenchymal stem cells are linked to genome instability and likely contribute to their eventual neoplastic transformation, including RSC development [44]. It will be of interest to examine strain differences in satellite cell response to injury and aging in the various RSC susceptible and resistant strains to determine whether this may influence strain variation in RSC growth.

We found several potential SNP differences in dysregulated transcripts in tumor samples that may be important for RSC development. The use of mouse RSC models to study microarray expression differences between neoplastic and normal tissues is well established and resulted in the discovery of several important potential therapeutic targets [45]. Analyzing tumor gene expression profiles can aid in the development of identification tools for tumor subclasses as well as identification of potential targets for intervention [33]. The combination of human tumor analysis and new animal models will produce a deeper understanding of RSC development, and will hopefully result in new therapeutic courses of action [33]. Our studies provide a new mouse strain, A/J, to the growing number of potential tools for dissecting the underlying genetic mechanisms responsible for susceptibility to APT RSC development.

## Materials and Methods

### Mice and tissue collection

A/J (JR# 646), BALB/cJ (JR# 651), SJL/J (JR# 686), and BALB/cByJ (JR# 1026) wild type mice were obtained from The Jackson Laboratory (Bar Harbor, ME). All studies were done with the approval of The Jackson Laboratory Institutional Animal Care and Use Committee (06005). Mice were maintained in conventional barrier facilities at 24±2°C and 51±7% relative humidity, housed in 333.6 cm^2^ maxi-miser Duplex II cages (Thoren caging systems, Hazleton, PA) with pine shavings, exposed to a 14 h light/10 h dark cycle, allowed free access to sterilized acidified water (pH 2.8–3.2), and fed autoclaved NIH-31 Rat & Mouse 5K54 Lab Diet® (PMI Nutrition International, St. Louis, MO) *ad libitum*. The health status of each animal room is evaluated every 13 weeks. This includes necropsy of representative clinically normal mice, culture of feces for pathogenic bacteria, fecal examinations for parasitic ova, serological screening for major mouse pathogens, and culture of selected organs for pathogenic bacteria (current status of our colonies can be found at this web site address: http://jaxmice.jax.org/html/health/quality_control.shtml#Animalhealth). Preventive medicine programs also include microbiological monitoring of sterilizers, culture of environmental surfaces for coliforms, monitoring animal water for pH or residual chlorine, and culture of feed ingredients.

Mice were maintained in four groups. One group of 65 females and 35 males was used to determine lifespan. Three additional groups consisted each of 15 females and 15 males that were necropsied at 6, 12, and 20 months of age [11], [12]. Detailed histopathological phenotyping was done on each mouse [46]. Moribund mice near death were submitted for histologic examination from all groups. Criteria for euthanasia approved by our Institutional Animal Care and Use Committee were as follows: not responsive to stimuli, slow respiration, cold to the touch, hunched with matted fur, sudden weight loss, failure to eat and drink, prominent appearing ribs and spine, and/or sunken hips. Mice were euthanized by CO_2_ asphyxiation. Tissues were either fixed by immersion using acid alcohol formalin, snap frozen in liquid nitrogen for DNA extraction, or snap frozen in RNAlater and stored at −80C for gene expression studies [46].

### Histological Analysis and Immunohistochemistry

Tissues for both routine histology and immunocytochemistry were fixed overnight in Feketes acid-alcohol-formalin solution (61% ethanol, 3.2% formaldehyde, 0.75N acetic acid), transferred to 70% ethanol, processed routinely, embedded in paraffin, sectioned at 5–6 µm, placed on microscope slides (Superfrost/Plus Fisherbrand, Pittsburgh, PA) and stained with hematoxylin and eosin (H&E) for routine histopathologic analysis. In addition, serial representative sections were stained with Bodian, phosphotungstic acid hematoxylin, and iron hematoxylin to demonstrate cross striations characteristic of skeletal muscle, a classical diagnostic criteria for RSC [8].

Triple immunofluorescence labeling was performed to evaluate markers of undifferentiated myoblasts and to rule out smooth muscle. Slides were deparaffinized in xylene and dehydrated using graded ethanols. Slides were boiled in 0.01 M sodium citrate/0.005% Tween 20 (pH 6.0) for 10 minutes for antigen retrieval, cooled to room temperature, rinsed 3 times in phosphate buffered saline (PBS, and incubated for 1 hr in blocking serum (0.3% Triton-X, 5% NGS or nonfat dry milk) in PBS). Excess blocking serum was removed and slides were incubated overnight at 4°C with primary antibodies (1∶250 anti-sarcomeric actin, mouse monoclonal IgM, Sigma; 1∶500 anti-smooth muscle actin, mouse monoclonal IgG_2a_, Sigma, St. Louis, MO; 1∶500 anti-myogenin, mouse monoclonal IgG_1_, BD Pharmingen, San Diego, CA) diluted in blocking serum. Myogenin was found to be the best marker for RSC immunohistochemistry [47]. Secondary antibodies were Alexa-conjugated fluorochromes 1∶500 IgM-546, 1∶1000 IgG_2a_-488, 1∶500 IgG_1_-647 (Molecular Probes, Carlsbad, CA).

### Gene Expression Studies

Four rhabdomyosarcomas (∼450 day old male A/J mice) and 8 nonneoplastic skeletal muscle samples (425–550 day old male A/J mice) were used. RNA was isolated from normal skeletal muscles or RSC by our Institutional Gene Expression Service. Briefly, tissues were stored in RNAlater® (Ambion Austin, TX) per manufacturer's instructions and homogenized in Trizol® Reagent (Invitrogen, Carlsbad, CA). Total RNA was isolated by standard Trizol methods, and quality was assessed using a 2100 Bioanalyzer instrument (Agilent, Santa Clara, CA). Total RNA was then treated with RNase-free DNase I (Qiagen, Valencia, CA) according to the manufacturer's protocol. Total RNA (1 µg) was reverse transcribed employing standard random hexamer priming methods and Superscript III enzyme (Invitrogen, Carlsbad, CA) according to the manufacturer's protocols. Primary RSC tissue samples were stored in RNALater prior to RNA extraction. Total RNA was isolated using Trizol reagent with manufacturer's standard protocols. RNA samples were subsequently processed for hybridization to Illumina Mouse Bead Arrays 6 V1.1 (Illumina, Inc., San Diego, CA). After microarray scanning, raw intensity values were quantile normalized and log^2^ transformed before fitting a model. The ANOVA model for normalized data was y ∼ Genotype. P values from 1000 permutations and fold change between mutant and wild type groups for each gene were calculated. To control false discovery rate, q values were also calculated for each gene. All analyses were done using R/Maanova 1.4.1. Relative fold changes were determined in RSC samples relative to normal skeletal muscle controls. Results for significantly altered transcripts were analysed using the Ingenuity Pathway Analysis® software (http://www.ingenuity.com/). The raw data generated was deposited in a MIAME compliant database (http://www.ncbi.nlm.nih.gov/geo/query/acc.cgi?acc=GSE29775; accession # GSE29775).
